# Monitoring for bovine arboviruses in the most southwestern islands in Japan between 1994 and 2014

**DOI:** 10.1186/s12917-016-0747-z

**Published:** 2016-06-24

**Authors:** Tomoko Kato, Tohru Yanase, Moemi Suzuki, Yoshito Katagiri, Kazufumi Ikemiyagi, Katsunori Takayoshi, Hiroaki Shirafuji, Seiichi Ohashi, Kazuo Yoshida, Makoto Yamakawa, Tomoyuki Tsuda

**Affiliations:** Kyushu Research Station, National Institute of Animal Health, NARO, 2702 Chuzan, Kagoshima, 891-0105 Japan; Okinawa Prefectural Institute of Animal Health, 1-24-29 Kohagura, Naha, Okinawa 900-0024 Japan; Yaeyama Livestock Hygiene Service Center, 1-2 Miyara, Ishigaki, Okinawa 907-0022 Japan; Viral Disease and Epidemiology Research Division, National Institute of Animal Health, NARO, 3-1-5 Kannondai, Tsukuba, Ibaraki 305-0856 Japan; Exotic Disease Research Station, National Institute of Animal Health, 6-20-1 Josuihoncho, Kodaira, Tokyo, 187-0222 Japan; National Institute of Animal Health, NARO, 3-1-5 Kannondai, Tsukuba, Ibaraki 305-0856 Japan

**Keywords:** Arbovirus, Bovine ephemeral fever, Cattle, *Culicoides* biting midges, Congenital abnormality, Epizootic hemorrhagic disease, Orbivirus, Orthobunyavirus, Rhabdovirus, Serosurveillance

## Abstract

**Background:**

In Japan, epizootic arboviral infections have severely impacted the livestock industry for a long period. Akabane, Aino, Chuzan, bovine ephemeral fever and Ibaraki viruses have repeatedly caused epizootic abnormal births and febrile illness in the cattle population. In addition, Peaton, Sathuperi, Shamonda and D’Aguilar viruses and epizootic hemorrhagic virus serotype 7 have recently emerged in Japan and are also considered to be involved in abnormal births in cattle. The above-mentioned viruses are hypothesized to circulate in tropical and subtropical Asia year round and to be introduced to temperate East Asia by long-distance aerial dispersal of infected vectors. To watch for arbovirus incursion and assess the possibility of its early warning, monitoring for arboviruses was conducted in the Yaeyama Islands, located at the most southwestern area of Japan, between 1994 and 2014.

**Results:**

Blood sampling was conducted once a year, in the autumn, in 40 to 60 healthy cattle from the Yaeyama Islands. Blood samples were tested for arboviruses. A total of 33 arboviruses including Akabane, Peaton, Chuzan, D’ Aguilar, Bunyip Creek, Batai and epizootic hemorrhagic viruses were isolated from bovine blood samples. Serological surveillance for the bovine arboviruses associated with cattle diseases in young cattle (ages 6–12 months: had only been alive for one summer) clearly showed their frequent incursion into the Yaeyama Islands. In some cases, the arbovirus incursions could be detected in the Yaeyama Islands prior to their spread to mainland Japan.

**Conclusions:**

We showed that long-term surveillance in the Yaeyama Islands could estimate the activity of bovine arboviruses in neighboring regions and may provide a useful early warning for likely arbovirus infections in Japan. The findings in this study could contribute to the planning of prevention and control for bovine arbovirus infections in Japan and cooperative efforts among neighboring countries in East Asia.

**Electronic supplementary material:**

The online version of this article (doi:10.1186/s12917-016-0747-z) contains supplementary material, which is available to authorized users.

## Background

Arthropod-borne viruses (arboviruses) are transmitted by hematophagous arthropod vectors, such as mosquitoes, ticks and *Culicoides* biting midges. Nearly 500 arboviruses have been recorded so far and some of them can seriously harm animal health [1]. Arbovirus infections often affect wide regions in a short period of time. The infected vectors are easily disseminated by air streams [2, 3], and their long-distance migrations (hundreds of kilometers) have been successfully estimated [4–7]. The recent emergence of bluetongue and Schmallenberg virus (SBV) infection in northern Europe demonstrated such rapid and wide expansion and caused huge economic damage to the livestock industry of countries that had been previously free from these arboviral infections [8, 9].

In Japan, epizootic abortion, stillbirth, premature birth and congenital malformations in cattle caused by arboviruses have severely impacted the livestock industry for a long period [10]. Akabane virus (AKAV) and Aino virus (AINOV) of the genus *Orthobunyavirus* in the family *Bunyaviridae* is principally associated with recurring epizootics of abnormal births [10–12]. It was estimated that approximately 42,000 abnormal calves caused by AKAV were born during the largest outbreak between 1972 and 1975. Chuzan virus (CHUV) of the genus *Orbivirus* in the family *Reoviridae* is also known to be an etiological agent of congenital abnormalities in cattle [13, 14]. In recent years, bovine encephalomyelitis caused by postnatal AKAV infection reemerged in Japan after a 21-year absence [15, 16]. Furthermore, incursions of Peaton virus (PEAV), Sathuperi virus (SATV) and Shamonda virus (SHAV) of the genus *Orthobunyavirus* were confirmed in Japan in the past 16 years [17–19]. Although these viruses potentially have teratogenicity in ruminants, little is known about their pathogenicity [20, 21]. As well as CHUV, D’Aguilar virus (DAGV) of the genus *Orbivirus* is a member of Palyam virus (PALV) group and has repeatedly been isolated in Japan since 1987 [22, 23]. Its etiological role in epizootic congenital abnormalities in cattle between 2001 and 2002 was serologically confirmed with colostrum-free calves. Bovine ephemeral fever virus (BEFV) of the genus *Ephemerovirus* in the family *Rhabdoviridae* causes a febrile illness in cattle and water buffalo, and is associated with reduction of milk production in dairy cattle and loss of condition in beef cattle [24]. The last occurrence of bovine ephemeral fever in mainland Japan was reported in 1991, but its periodic epizootics continue in the southwestern islands [25, 26]. Ibaraki virus (IBAV) is a strain of epizootic hemorrhagic disease virus (EHDV) serotype 2 of the genus *Orbivirus*, and difficulty swallowing is its main manifestation [27]. The contribution of EHDV serotype 7 (EHDV-7) to epizootic abortion and stillbirth in pregnant cows was also reported in the southern part of Japan in 1997 [28–30]. The above-mentioned arboviruses are principally transmitted by *Culicoides* biting midges [23, 31] while mosquitoes are strong candidates for BEFV vectors [24]. Because the vector activity ceases in winter, the overwintering of arboviruses is seemingly unrealistic in Japan. In fact, appearances of the same strain/genotype of arboviruses in consecutive years rarely happen in Japan [10, 32, 33]. Development of efficient commercial vaccines to AKAV, AINOV, CHUV, IBAV and BEFV has contributed to reducing diseases in cattle, but arbovirus infections still break out in unvaccinated cattle, and no effective prevention measures are prepared for newly emerged arboviruses.

The ideal climate for vector insects in the tropical zone permits arbovirus circulation through most of the year. Therefore, the lower-latitude regions are considered a potential source of arboviruses in the temperate zone, where vector insects are usually absent during the colder months. *Culicoides*- and mosquito-borne arboviruses are considered to be introduced from lower latitudes in Asia to Japan with the infected vectors carried by airstreams [10, 34]. Although former studies revealed the prevalence of several ruminant arboviruses in Southeast Asian countries [35–37], details of arbovirus activity remain unknown. The Yaeyama Islands in Okinawa Prefecture are located in the subtropical zone and are 100–200 km, 350–450 km, 700 km and 1000 km away from Taiwan, Southern China, Philippines and mainland Japan, respectively. Due to their geographic location, the islands may be well suited for playing a role as an early warning system for other parts of Japan. Indeed, it was suggested that previous epizootics of bovine ephemeral fever in the Yaeyama Islands were epidemiologically linked to those in Taiwan [25, 26, 38]. The enhanced surveillance of ruminant arboviruses in the bordering area could support the planning of preventive and control strategies for arbovirus infections in the remaining part of Japan. To monitor the annual incursion of arboviruses into the Yaeyama Islands, we conducted virus isolation from sentinel cattle and serosurveillance for the above-mentioned arboviruses in the cattle population for 20 years. This long-term monitoring indicates a high risk of incursion by multiple arboviruses from overseas to the Yaeyama Islands. We also discuss whether the monitoring in the islands could contribute to earlier detection of arbovirus activities prior to their spread in mainland Japan.

## Methods

### Study area

**Fig. 1:**
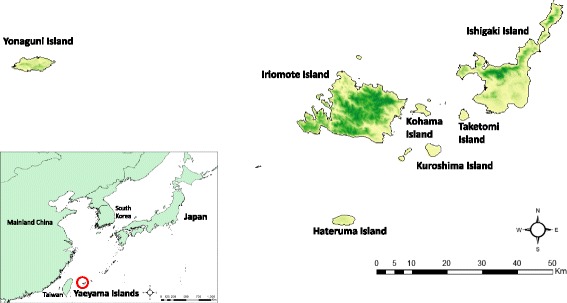
Geographical location of the study area. The maps were generated using Arc GIS 10 (Esri, Redlands, CA) based on data retrieved from DIVA-GIS and the National Land Numerical Information download service provided by Ministry of Land, Infrastructure, Transport and Tourism, Japan

The Yaeyama Islands comprise an archipelago located in the most southwestern part of Japan (24.00-24.67° N, 122.75-124.50°E) from Ishigaki Island to Yonaguni Island (Fig. 1). Approximately 36,000 beef cattle are reared through the islands, and over 70 % of this cattle population exists on Ishigaki Island, which is the second largest island in the archipelago. The climate of the Yaeyama Islands is subtropical; the annual mean temperature was 24 °C between 1994 and 2014; mean temperatures of the hottest and coldest months are about 29 °C and 19 °C, respectively. Annual precipitation was about 2200 mm and humidity ranged from 70 to 80 % during the study period. Blood sampling was continuously conducted in Ishigaki, Iriomote and Yonaguni Islands, but was optional in Taketomi, Kohama, Kuroshima and Hateruma Islands (Table 1).

**Table 1:** Blood samples used for virus isolation and neutralization tests

Year	Collection date	No. of blood samples	Blood collection sites (No. of blood samples by collection site: virus isolation/VNTs)
		Virus isolation/VNTs^b^	
1994	21.October – 7.November	58/0	Ishigaki (10/0), Taketomi (10/0), Kohama (10/0), Hateruma (10/0), Kuroshima (8/0), Yonaguni (10/0)
1995	7 – 17.November	50/49	Ishigaki (40/39), Yonaguni (10/10)
1996	17 – 28.October	50/30	Ishigaki (25/25), Iriomote (15/5), Yonaguni (10/0)
1997	22–31.October	60/49	Ishigaki (35/30), Iriomote (15/9), Yonaguni (10/10)
1998	10–11.November	50/39	Ishigaki (20/20), Iriomote (20/10), Yonaguni (10/9)
1999	16–18.November	50/40	Ishigaki (20/13), Iriomote (10/9), Kuroshima (10/9), Yonaguni (10/9)
2000	15–17.November	50/36	Ishigaki (20/13), Iriomote (10/9), Kuroshima (10/7), Yonaguni (10/7)
2001	12–13.November	40/39	Ishigaki (20/20), Yonaguni (20/19)
2002	25. November	50/48	Ishigaki (20/20), Iriomote (10/8), Kuroshima (10/10), Yonaguni (10/10)
2003	26–28.November	55/53	Ishigaki (20/19), Taketomi (10/10), Iriomote (15/14), Yonaguni (10/10)
2004^a^	-	-	-
2005	7–12. December	55/52	Ishigaki (10/9), Taketomi (10/10), Iriomote (12/12), Kuroshima (10/9), Yonaguni (13/12)
2006	27. November-8.December	50/45	Ishigaki (24/23), Iriomote (11/10), Kuroshima (10/7), Yonaguni (5/5)
2007	27. November-5.December	50/45	Ishigaki (26/23), Taketomi (3/3), Iriomote (17/16), Yonaguni (4/3)
2008	11. November-11.December	51/48	Ishigaki (21/20), Iriomote (8/7) Taketomi (4/4), Kohama (3/3), Hateruma (5/5), Kuroshima (5/4), Yonaguni (5/5)
2009	17–25.November	52/49	Ishigaki (15/15), Taketomi (7/7), Iriomote (10/9), Kohama (5/5), Kuroshima (5/5), Yonaguni (10/8)
2010^a^	-	-	-
2011	29. November-1.December	50/49	Ishigaki (25/24), Iriomote (20/20), Yonaguni (5/5)
2012	3–5.December	51/47	Ishigaki (21/19), Iriomote (21/20), Kohama (9/8)
2013	11.November-4.December	50/50	Ishigaki (11/11), Taketomi (3/3), Iriomote (11/11), Kohama (3/3), Hateruma (5/5), Kuroshima (7/7), Yonaguni (10/10)
2014	27.October-26.November	55/45	Ishigaki (12/12), Taketomi (3/2), Iriomote (11/5), Kohama (6/6), Hateruma (6/6), Kuroshima (10/8), Yonaguni (7/6)
Total		977/813	

^a^Sampling was not done in 2004 and 2010

^b^VNTs: virus neutralization tests

### Blood collection

Blood sampling was conducted once a year between the middle of October and beginning of December in 1994–2003, 2005–2009 and 2011–2014 (Table 1). Heparinized blood and serum samples were obtained from 40–60 healthy cattle. The blood samples were separated into plasma and blood cells by centrifugation and the blood cells were washed three times with phosphate buffered saline to eliminate the antibodies. The plasmas and blood cells were stored at −80 °C until virus isolation. The serum samples were kept at −20 °C until they were used for virus neutralization tests (VNTs). A total of 977 blood samples were subjected to arbovirus screening. To detect antibodies against arboviruses, VNTs were conducted for 813 serum samples harvested after 1995. The tested sera were selectively collected from cattle that were 6–12 months old and had only been alive for one summer. The blood samples were voluntarily collected by local veterinary officers for monitoring of arbovirus infections and were permitted to be used in this study from the animal health authority of Okinawa Prefecture.

### Virus isolation

The processed plasmas and blood cells were inoculated into monolayer cultures of baby hamster kidney (BHK-21) and hamster lung (HmLu-1) cells as described previously [23]. Briefly, cells were incubated into test tubes with Eagle’s minimum essential medium (MEM) (Nissui, Tokyo, Japan) supplemented with 0.295 % tryptose phosphate broth (Becton Dickinson and Company, Franklin Lakes, NJ, USA), 0.015 % sodium bicarbonate and 10 % of bovine serum overnight at 37 °C and were washed three times with Earl’s solution before inoculation. The cell cultures inoculated with blood samples were maintained in serum-free medium by rotation at 37 °C for 7 days and were collected if a cytopathic effect (CPE) was observed. Two further blind passages were conducted in the same manner if CPE was not observed.

### Dot immunobinding assay

Cytopathic agents in the cultured cells were characterized by a dot immunobinding assay (DIA) as described previously [23, 39]. Briefly, the supernatants of cell cultures showing CPE were blotted onto the Immobilon PVDF transfer membrane (Millipore, Billerica, MA, USA) using a slot blotting apparatus. The membrane was immersed in blocking buffer [8 % skim milk in Tris-buffered saline (TBS; 20 mM Tris–HCl pH 7.5, 0.15 M NaCl)] for more than 2 h and then reacted with monoclonal antibodies to the nucleocapsid protein and viral surface glycoprotein (Gc) of AKAV, the Gc protein of AINOV and PEAV, and mouse antisera to CHUV, IBAV and bluetongue virus for 1 h. The membrane was reacted with horseradish peroxidase-conjugated goat antibody to mouse IgG in blocking buffer for 30 min, and the immune complex was detected by color development with 0.027 % 3,3-diaminobenzidine tetrahydrochloride and 0.016 % H_2_O_2_ in TBS.

### RT-PCR and sequence analysis

Viral RNA was extracted from the supernatant of cell cultures showing CPE with the High Pure Viral RNA Kit (Roche Diagnostics, Mannheim, Germany). Group-specific RT-PCRs targeting the S RNA segment of orthobunyaviruses, segment 3 of PALV group viruses and segment 7 of EHDV were performed using the Titan One tube RT-PCR Kit (Roche Diagnostics) in accordance with previous studies [40, 41]. Specific detections of segment 2 of CHUV and DAGV by RT-PCR were conducted with the primer sets CHUVL2F-2/CHUVL2R-2 and DAGVL2F/DAGVL2R-2, respectively (Additional file 1) [23]. An isolate of EHDV was tested in an RT-PCR assay with the serotype-specific primer sets targeting segment 2 [41]. Sample denaturation at 94 °C for 4 min was performed for the orbivirus detection before cDNA synthesis. The cDNA synthesis was conducted at 50 °C for 30 min followed by 94 °C for 2 min. The PCR profile was 10 cycles of 94 °C for 30 s, 55 °C for 30 s and 68 °C for 45 s, followed by 25 cycles of 94 °C for 30 s, 55 °C for 30 s and 68 °C for 45 s, with the latter time increased by 5 s per cycle.

Genome RNA of an orbivirus that was unidentified by the above-mentioned methods was extracted from the infected cells using the TRIZOL LS Reagent (Life Technologies, Carlsbad, CA, USA). Amplification of the cDNA of segment 2 was performed by Full-Length Amplification of cDNAs (FLAC) as described previously [42]. In brief, double-stranded RNA was ligated to the anchor primer (5-15-1) with T4 RNA ligase (New England Bio Labs, Ipswich, MA, USA) at 4 °C overnight and separated in 1 % agarose gel by electrophoresis. Segment 2 was recovered from the gel and purified using the RNaid Kit with SPIN (Bio 101, Vista, CA, USA). First-strand cDNA synthesis was performed using the Superscript III First-Strand Synthesis System for RT-PCR (Life Technologies) in accordance with the manufacturer’s instructions. The cDNA was amplified using KOD-plus-ver.2 (TOYOBO, Osaka, Japan) in a reaction mixture under the following conditions: 30 cycles of 98 °C for 10 s, 62 °C for 30 s and 68 °C for 3.5 min.

The PCR products were purified with the QIAquick PCR Purification Kit (Qiagen, Hilden, Germany) and directly sequenced with the BigDye Terminator Cycle sequencing Kit v3.1 (Life Technologies) on the ABI 3100-Avanti Genetic Analyzer (Life Technologies). The nucleotide (nt) sequences were edited by DNASIS Pro Ver. 3.0 (Hitachi Solutions, Tokyo, Japan), and a sequence similarity search was conducted with the basic local alignment search tool (BLAST). Pairwise nt sequence identities were calculated with GENETYX software ver. 10 (GENETYX, Tokyo, Japan).

### Virus neutralization test

Virus neutralization tests with AKAV OBE-1, AINOV JaNAr28, PEAV KSB-1/P/06, SATV KSB-2/C/08, SHAV KSB-6/C/02, BEFV YHL, CHUV C31, DAGV KSB-29/E/01, IBAV No.2 and EHDV-7 KSB-14/E/97 were performed. After heat inactivation at 56 °C for 30 min, bovine sera were serially diluted twofold in serum-free Eagle’s MEM containing 10 μg/ml gentamicin sulfate from 1:2 to 1:64 in the 96-well microplates. Fifty microliters of serum dilution was mixed with an equal volume of virus inoculum containing 100 × the 50 % tissue culture infective doses and incubated at 37 °C under 5 % CO_2_ for 1 h. Then, 100 μl of the suspension of HmLu-1 cells in GIT medium (Wako Pure Chemical Industries, Ltd., Osaka, Japan) was added into each well and incubated at 37 °C under 5 % CO_2_ for 7 days. The antibody titer was calculated as the reciprocal of the highest serum dilution inhibiting the CPE. Samples were deemed positive if they had neutralizing antibodies to the viruses in at least a dilution of 1/8.

## Results

### Isolation and identification of arboviruses from bovine blood samples

A total of 33 arbovirus isolates were obtained from bovine blood samples during the study period (Table 2). Some of the isolated viruses had been genetically analyzed in previous studies [22, 32, 33]. Nine isolates obtained in 1994, 1998 and 2001 were identified as AKAV by DIA. Four PEAV isolates were also found in the viruses obtained in 2001 and 2009. Eighteen isolates were reacted with the polyclonal antibodies against CHUV in DIAs and the group-specific and strain-specific RT-PCRs sorted themselves into CHUV (6 isolates in 1998, 2002 and 2006), DAGV (5 isolates in 2000, 2006 and 2012) and an unidentified PALV group virus (7 isolates in 2008 and 2009). Segment 2 of ON-1/E/08, which is an isolate of the unidentified PALV group viruses, was amplified by the FLAC method. The whole sequence of segment 2 (GenBank accession no. AB973440) showed the highest identity with Bunyip Creek virus (BCV) CSIRO58 (90.8 % nt and 94.9 % aa identities). The other 6 isolates of the PALV group viruses tested positive by RT-PCR with the BCV segment 2-specific primer pair. DIA and the group-specific RT-PCRs revealed that a 2003 isolate designated as ON-1/E/03 belongs to EHDV. High levels of similarity (82–84 % nt identity) in segment 7 between ON-1/E/03 (GenBank accession No. LC066302) and other EHDV strains were also revealed. However, ON-1/E/03 tested negative by RT-PCRs with the serotype-specific primer sets. The remaining isolate obtained in 1994 could not be identified by the serological tests. However, detailed genetic analyses in a previous study determined the isolate as Batai virus (BATV) of the genus *Orthobuyavirus* [43].

**Table 2:** Arboviruses isolated in the Yaeyama Islands from 1994 to 2014

				No. of isolates		
Virus	Year	Date	Place	Plasma	Blood cells	Sensitive cell line
Genus *Orthobunyavirus*						
Akabane virus	1994	25. October	Ishigaki	3		BHK-21, HmLu-1
	1998	11. November	Iriomote	2	2	BHK-21, HmLu-1
	2001	12. November	Ishigaki	2		BHK-21, HmLu-1
Peaton virus	2001	13. November	Yonaguni		2	BHK-21
	2009	24. November	Ishigaki	1	1	BHK-21
Batai virus	1994	27. October	Yonaguni		1	BHK-21, HmLu-1
Genus *Orbivirus*						
Chuzan virus	1998	10. November	Ishigaki		1	BHK-21
	2002	25. November	Yonaguni		1	BHK-21
	2006	27. November - 8. December	Iriomote, Ishigaki		4	BHK-21
D’ Aguilar virus	2000	15. November	Ishigaki		2	BHK-21, HmLu-1
	2006	8. December	Ishigaki		1	BHK-21
	2012	3, 4. December	Ishigaki		2	BHK-21
Bunyip Creek virus	2008	11. December	Yonaguni	1	2	BHK-21, HmLu-1
	2009	18, 24. November	Ishigaki, Taketomi		4	BHK-21
EHD^a^ virus	2003	27. November	Taketomi		1	BHK-21

^a^ *EHD*, epizootic hemorrhagic disease

### Detection of antibodies against bovine arboviruses

**Fig. 2:**
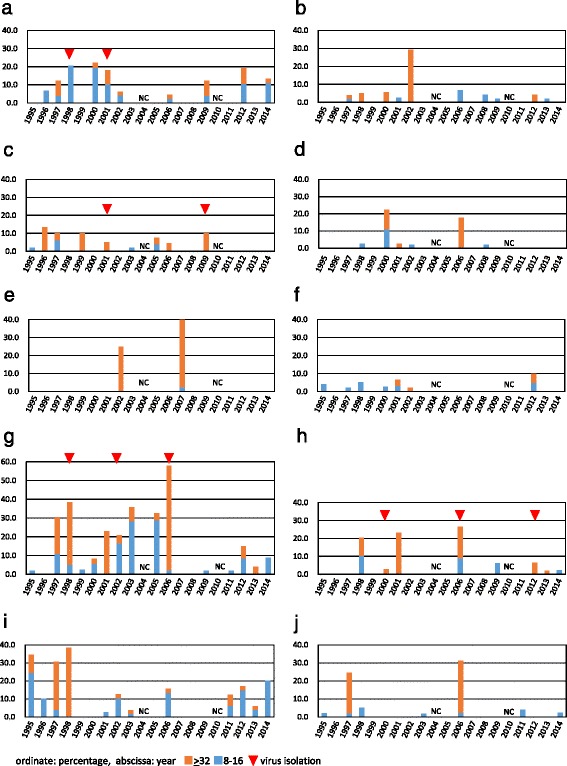
Seroprevalence of bovine arboviruses including AKAV (**a**), AINOV (**b**), PEAV (**c**), SATV (**d**), SHAV (**e**), BEFV (**f**), CHUV (**g**), DAGV (**h**), IBAV (**i**) and EHDV-7 (**j**) in sentinel cattle in the Yaeyama Islands. Down-pointing triangle indicates the year of isolation of each virus. NC: not conducted

All arboviruses that were investigated in this study were observed as present in cattle blood in the Yaeyama Islands between 1995 and 2014 (Fig. 2). Neutralizing antibodies to the above-mentioned arboviruses were often detected in the 4-fold diluted serum. These results may indicate infection by another closely related virus [44–46] or the presence of maternally derived antibodies included in colostrum [47, 48]. Also, non-specific reactivity due to the low dilution of the sera in the VNTs could not be ruled out. Therefore, neutralizing antibody titers < 1:8 were defined as negative. Neutralizing antibodies to AKAV, AINOV, CHUV and IBAV were detected in tested cattle for more than 10 years during the study period. The seroprevalence varied a great deal according to the tested viruses (4.4–22.2 % for AKAV; 2.0–29.2 % for AINOV; 2.0–57.8 % for CHUV and 2.6–38.5 % for IBAV) and the year (Additional file 2). PEAV, BEFV, DAGV and EHDV-7 were each observed in 7–9 years. Neutralizing antibodies against SHAV and SATV were detected in 2–6 years. Serum samples collected in the Yaeyama Islands in 1994 were attempted to detect the antibodies against AKAV and BATV. A certain level of seroprevalence for both viruses (10.3 % for AKAV and 50.0 % for BATV) was observed in examined cattle (data not shown).

Arboviruses were frequently detected in the blood of cattle on Ishigaki and Iriomote Islands, but only occasionally in those of the other islands (Table 3). However, the seroprevalence in Yonaguni Island was sometimes independent of those in Ishigaki and Iriomote Islands (e.g. against PEAV in 2001 and 2006). Synchronization of seroprevalence between Ishigaki Island and surrounding islands, Taketomi, Kohama, Kuroshima and Hateruma, was generally observed during the study period.

**Table 3:** Seroprevalence of arboviruses by each island between 1995 and 2014

Year	AKAV			AINOV			PEAV			SATV			SHAV			BEFV			CHUV			DAGV			IBAV			EHDV-7		
	IS	IR	YO	IS	IR	YO	IS	IR	YO	IS	IR	YO	IS	IR	YO	IS	IR	YO	IS	IR	YO	IS	IR	YO	IS	IR	YO	IS	IR	YO
1995		—			—		●	—			—			—			—	●	●	—			—		●	—	●	●	—	
1996	●		—			—	●	●	—			—			—			—			—			—	●	●	—			—
1997	●		●	●			●									●			●	●					●	●		●		
1998	●	●		●						●								●	●	●		●	●		●	●	●	●	●	
1999																					●									
2000		●	●	●						●	●						●			●			●							
2001	●	—	●		—	●		—	●	●	—			—		●	—		●	—	●	●	—	●	●	—			—	
2002		●	●	●	●						●			●	●		●		●	●	●					●	●			
2003								●											●	●	●				●	●		●		
2004	—	—	—	—	—	—	—	—	—	—	—	—	—	—	—	—	—	—	—	—	—	—	—	—	—	—	—	—	—	—
2005								●	●										●	●	●									
2006	●			●	●				●	●	●								●	●	●	●	●		●	●		●	●	●
2007													●	●	●															
2008						●				●																				
2009	●	●					●	●												●			●							
2010	—	—	—	—	—	—	—	—	—	—	—	—	—	—	—	—	—	—	—	—	—	—	—	—	—	—	—	—	—	—
2011																				●						●	●		●	
2012	●	●	—	●		—			—			—			—	●	●	—	●	●	—	●		—	●	●	—			—
2013					●															●			●				●			
2014	●	●																		●	●				●	●		●		

*IS* Ishigaki Island, *IR* Iriomote Island, *YO* Yonaguni Island

●: Seroprevalence was detected. —: Blood sampling was not conducted

## Discussion

In Japan, bovine diseases associated with AKAV, AINOV, CHUV, IBAV and BEFV are notifiable diseases under the domestic animal infectious disease control law, and nationwide serosurveillance for these arboviruses with sentinel cattle has been conducted since 1985 [47, 49, 50]. AKAV activity has been observed on mainland Japan almost every year. Also, on the Yaeyama Islands, AKAV was frequently observed and was sometimes linked with occurrences of disease. The isolated viruses in 1994 were genetically undistinguishable from AKAVs isolated on mainland Japan the following year [32, 33]. The finding indicated that the earlier detection of AKAV activity in the Yaeyama Islands could be available in some cases prior to its spread in the mainland. Although the AINOV isolation was not made in this study, its prevalence in the Yaeyama Islands was observed by serological surveillance. The epizootics of abnormal births caused by AINOV occurred in mainland Japan, in 1998–1999, 2002–2003 and 2005–2006 seasons. In contrast, the seroprevalence in the Yaeyama Islands was concurrently found in 1998 and 2002. Although AINOV has not been detected in the mainland since 2006, neutralizing antibodies against AINOV were continuously detected in the islands, indicating that AINOV is still active in the lower latitudes in Asia and its reemergence in the mainland is highly possible.

On mainland Japan, the last occurrence of congenital abnormalities by CHUV was reported in 2000 and, since then, CHUV has not been detected. However, the CHUV activity in the neighboring regions could be estimated from the frequent sero-detection and the virus isolations in the Yaeyama Islands. Although no clinical case was reported, the activity of DAGV had been detected several times in the Yaeyama Islands since 1991. It is noteworthy that a DAGV isolate in Ishigaki Island in 2000 shared 99.5 % nt identity in segment 2 with DAGV isolated in mainland Japan during the 2001 outbreak [22]. Furthermore, DAGV returned to the mainland in 2013 after its activity was observed in the Yaeyama Islands in the previous year [23].

Neutralizing antibodies to BEFV were present in the tested cattle in 2001 and 2012 when outbreaks of bovine ephemeral fever occurred. Low seroprevalences (2.0–5.1 %) of BEFV were also detected without clinical cases in 1995, 1997, 1998, 2000 and 2002, suggesting that the incursion and small-scale circulation of BEFV often occurs in the Yaeyama Islands. This also reflects the pattern of frequent epizootics of BEFV in neighboring regions, such as Taiwan and mainland China, in recent years [38, 51–53].

The resurgence of IBAV in the mainland was confirmed in 2013 after a 26-year absence [30]. However, the neutralizing antibodies to IBAV have been frequently detected in the Yaeyama Islands during the study period. Although the clinical evidence and the virus isolation were reported in Taiwan and Korea, respectively [54, 55], there is currently little information on this virus outside of Japan. It should be noted that IBAV caused an extensive outbreak of disease in cattle in Japan in 1959–1960 and resulted in the deaths of over 4000 cattle [27]. The epizootic of EHDV-7 once occurred in the mainland in 1997 [28]. Relatively high seroprevalences (24.5 and 31.1 %) of the virus were detected in the Yaeyama Islands in both 1997 and 2006. Although the serological surveillance suggests the continuous circulation of EHDV-7 in Asian countries, the epizootic abortion and still birth in cattle caused by the virus has not been recorded outside of Japan. However, this virus poses a serious potential threat to the livestock industry because no effective vaccine is currently available. Lower neutralization antibodies (≤1:8) to each of IBAV and EHDV-7 are often detected, probably due to the cross-reactivities with heterogeneous serotypes of EHDV [46]. The possible incursions of other EHDV serotypes into the cattle population in the Yaeyama Islands should be investigated in the future.

The pathogenicities of PEAV, SATV, and SHAV in cattle remain uncertain. However, colostrum-free calves with congenital abnormalities sometimes carry neutralizing antibodies to these viruses, suggesting their teratogenicity [17–19]. Although the detection of PEAV in Japan was first reported in 1999 [17], retrospective analysis for an unassigned virus from Ishigaki Island revealed that PEAV was already introduced as early as 1987 [56]. Repeated isolation of PEAV in the Yaeyama Islands, other islands in Okinawa Prefecture and mainland Japan also may link to its widespread circulation in Asian countries. Seroprevalences for SATV and SHAV were lower than those of the other arboviruses. However, the surveillance in the Yaeyama Island agreed with the recent incursion of these viruses to mainland Japan [18, 19, 21]. Although suspected clinical cases caused by SATV and SHAV have been very few in Japan, their close relationship with SBV has been clearly indicated by previous studies [21, 57, 58]. It is uncertain whether Japanese strains of SATV and SHAV, as well as SBV, maintain high pathogenicity to ruminants. However, the possible presence of SATV and SHAV should be considered to avoid misdiagnosis of SBV infection in the regions commonly affected by insect-borne arboviruses.

Attempts at virus isolation proved the incursions of BATV, BCV and an EHDV isolate to the Yaeyama Islands. Serological surveillance in 1994 and 1995 also revealed the transient incursion of BATV in 1994 (data not shown). It was reported that BATV occasionally causes a febrile illness in humans and ruminants [59]. It was noteworthy that BATV probably provided a genome segment to Ngari virus, associated with severe hemorrhagic disease in humans [43, 60]. Epizootic activity of BATV is supposed to continue in Asian countries [43, 61] and thus its involvement in human and animal diseases should be monitored hereafter. Although BCV infection in the cattle population was evident in this and previous studies [62, 63], its contribution to cattle illness is not yet clear. The BCV isolation was made in mainland Japan in 2009, one year after the initial isolation in the Yaeyama Islands [23]. The co-circulation of BCV and other PALVs, such as CHUV and DAGV, potentially generate reassortants between these viruses. Because the reassortment may cause changes in viral pathogenicity as well as viral genetic and serological properties, the emergence of reassortants among PALVs should be monitored. The EHDV isolate was obtained from bovine blood in 2003. Unfortunately, serotyping for the isolated EHDV could not be concluded by the current serotype-specific RT-PCRs [41]. The existence of multiple serotypes of EHDV makes the diagnostics of EHDV-related disease more complicated. To our knowledge, at least three serotypes of EHDV have been detected in Japan so far [30]. Further investigation, such as the use of FLAC, should be conducted to investigate the detailed properties of the virus to develop proper diagnostic systems in this region.

Annual seroprevalence patterns differed among the islands. Several factors could contribute to this result. The sampling size and cattle population on Ishigaki Island are significantly larger than those on other islands, probably resulting in the higher frequency of the virus and detection of neutralizing antibodies. Because the distance between Ishigaki Island and surrounding islands including Iriomote, Taketomi, Kohama and Kuroshima Islands is not so far (maximally 15 km apart), wind dispersal of infected vectors likely occurs between the islands. However, Yonaguni Island is approximately 65–110 km from the other islands in the archipelago. This geographical feature might cause independent epizootics there. In addition, Yonaguni Island is the part of Japan closest to Taiwan and mainland China, and thus more sensitive detection of arbovirus activities in adjacent regions could be conducted in some cases. Although the source of arboviruses remains unknown, long-distance migration of the infected insect vectors by air flows is the most probable route by which bovine arboviruses are introduced to the Yaeyama Islands. Atmospheric dispersal modeling would be useful to estimate plausible incursion events and possible source sites of infected vectors.

It is of interest that fewer clinical cases have been reported in the Yaeyama Islands, despite the frequent incursion of ruminant-pathogenic arboviruses. Most cows kept for reproduction in the islands probably gain humoral immunity to the viruses by vaccination or natural infection before their pregnancy. Therefore, the abnormal births might be prevented in most cases if the teratogenic arboviruses were introduced to the islands. Also, constant and high seroprevalence in the cattle population might limit acute and massive epizootics of arboviruses.

Because viremia of bovine arboviruses is generally short, the occasion of isolation is highly limited from sentinel cattle [23]. Therefore, the frequency of blood sampling in this study might not be enough for effective virus isolation. *Culicoides* biting midges are still active in the Yaeyama Islands between October and December [64] and thus the viral transmission possibly occurred after the blood samplings. To obtain the conclusive seroprevalence in each season, additional sampling from the sentinel cattle should have be conducted few months later. It would be necessary to modify the frequency and period of blood sampling for future monitoring in this area.

Too often ruminant arbovirus diseases are ignored and neglected in tropical countries because they have not yet impacted the livestock industry on the surface. However, these diseases might have caused continuous, but unrecognized losses in these countries. Further investigation regarding the ruminant arboviruses will be necessary to assess their impact on the livestock industry in the regions where they persist. Moreover, environmental change through global warming will enhance the virus spread from the tropical to the temperate zone [65]. Monitoring for exotic arboviruses increases in importance in the high-latitude regions where such viruses would do more serious damage to the livestock industry. The Yaeyama Islands were identified as a high-risk zone for arbovirus incursions in this study and thus would be an ideal point for monitoring.

## Conclusions

We showed that long-term monitoring in the Yaeyama Islands could estimate the activity of bovine arboviruses in neighboring regions and may provide a useful early warning of risk of arbovirus infections in Japan. The findings in this study could contribute to planning of prevention and control for bovine arbovirus infections in Japan and also to beneficial information-sharing on monitoring efforts among neighboring countries. Enhancement of collaborative work in this region should be essential to reduce the livestock loss caused by arbovirus infections.

## Abbreviations

AINOV, Aino virus; AKAV, Akabane virus; BATV, Batai virus; BCV, Bunyip Creek virus; BEFV, bovine ephemeral fever virus; CHUV, Chuzan virus; CPE, cytopathic effects; DAGV, D’Aguilar virus; DIA, dot immunobinding assay; EHDV, epizootic hemorrhagic disease virus; FLAC, Full-length Amplification of cDNAs; IBAV, Ibaraki virus; MEM, minimum essential medium; PALV, Palyam virus; PEAV, Peaton virus; SATV, Sathuperi virus; SBV, Schmallenberg virus; SHAV, Shamonda virus; VNT, virus neutralization test

## Additional files

Additional file 1: Primers used for RT-PCR and sequencing in this study. (XLSX 12 kb) Additional file 2: Seropositive rate to arboviruses in the Yaeyama Islands from 1995 to 2014. (XLSX 11 kb)
